# Implication of backward contact tracing in the presence of overdispersed transmission in COVID-19 outbreaks

**DOI:** 10.12688/wellcomeopenres.16344.3

**Published:** 2021-03-31

**Authors:** Akira Endo, Centre for the Mathematical Modelling of Infectious Diseases COVID-19 Working Group, Quentin J. Leclerc, Gwenan M. Knight, Graham F. Medley, Katherine E. Atkins, Sebastian Funk, Adam J. Kucharski

**Affiliations:** 1 Department of Infectious Disease Epidemiology, London School of Hygiene & Tropical Medicine, London, WC1E 7HT, UK; 2 The Alan Turing Institute, London, NW1 2DB, UK; 3 Centre for the Mathematical Modelling of Infectious Diseases, London School of Hygiene & Tropical Medicine, London, WC1E 7HT, UK; 4 Department of Global Health and Development, London School of Hygiene & Tropical Medicine, London, WC1E 7HT, UK; 5 Centre for Global Health Research, Usher Institute, University of Edinburgh, Edinburgh, EH16 4UX, UK

**Keywords:** COVID-19, SARS-CoV-2, contact tracing, backward tracing, overdispersion

## Abstract

**Introduction:** Contact tracing has the potential to control outbreaks without the need for stringent physical distancing policies, e.g. civil lockdowns. Unlike forward contact tracing, backward contact tracing identifies the source of newly detected cases. This approach is particularly valuable when there is high individual-level variation in the number of secondary transmissions (overdispersion).

**Methods:** By using a simple branching process model, we explored the potential of combining backward contact tracing with more conventional forward contact tracing for control of COVID-19. We estimated the typical size of clusters that can be reached by backward tracing and simulated the incremental effectiveness of combining backward tracing with conventional forward tracing.

**Results:** Across ranges of parameter values consistent with dynamics of SARS-CoV-2, backward tracing is expected to identify a primary case generating 3-10 times more infections than a randomly chosen case, typically increasing the proportion of subsequent cases averted by a factor of 2-3. The estimated number of cases averted by backward tracing became greater with a higher degree of overdispersion.

**Conclusion: **Backward contact tracing can be an effective tool for outbreak control, especially in the presence of overdispersion as is observed with SARS-CoV-2.

## Introduction

Isolation of symptomatic cases and tracing and quarantine of their contacts is a staple public health control measure, and has the potential to prevent the need for stringent physical distancing policies that result in detrimental impacts on the society (e.g., civil lockdowns) ^1, 2^. Contact tracing is typically triggered by a confirmed index case identified via symptom-based surveillance. Contacts of this index case are identified via interviews by public health officials (manual contact tracing) or by tracking proximity records on digital devices (digital contact tracing), and asked to quarantine in order to prevent further transmissions.

Contact tracing often targets ‘downstream’ individuals, who may have been infected by the index case (‘forward tracing’); i.e. those who have been in contact with the index case after the index case likely became infectious (often assumed as 2 days before illness onset for COVID-19 ^3, 4^). However, ‘backward tracing’ can also be used to identify the upstream primary case who infected the index case (or a setting or event at which the index case was infected) by retracing history of contact to the likely point of exposure up to the upper bound of the incubation period. For example, contact history of 14 days prior to symptom onset is collected in Japan, where backward tracing has been operated from the early phase of the COVID-19 outbreak ^5, 6^. If this primary case is identified, a larger fraction of the transmission chain can be detected by forward tracing each of the contacts of this primary case.

Unlike forward tracing, backward tracing is more effective when the number of onward transmissions is highly variable, because index cases are disproportionately more likely to have been generated by primary cases who also infected others (an example of the “friendship paradox” ^7– 9^). Because there is evidence that the number of secondary transmissions of SARS-CoV-2 per case exhibits substantial individual-level variation (i.e. overdispersion), often resulting in so-called superspreading events ^10– 12^, a large proportion of infections may be linked to a small proportion of original clusters. As a result, finding and targeting originating clusters in combination with reducing onwards infection may substantially enhance the effectiveness of tracing methods ^9, 13, 14^.

In the present study, using a simple branching process model, we explore the incremental effectiveness of combining ‘backward’ tracing with conventional ‘forward’ tracing in the presence of overdispersion in SARS-CoV-2 transmission.

## Methods

### Overdispersion and the coverage of contact tracing

**Figure 1.**
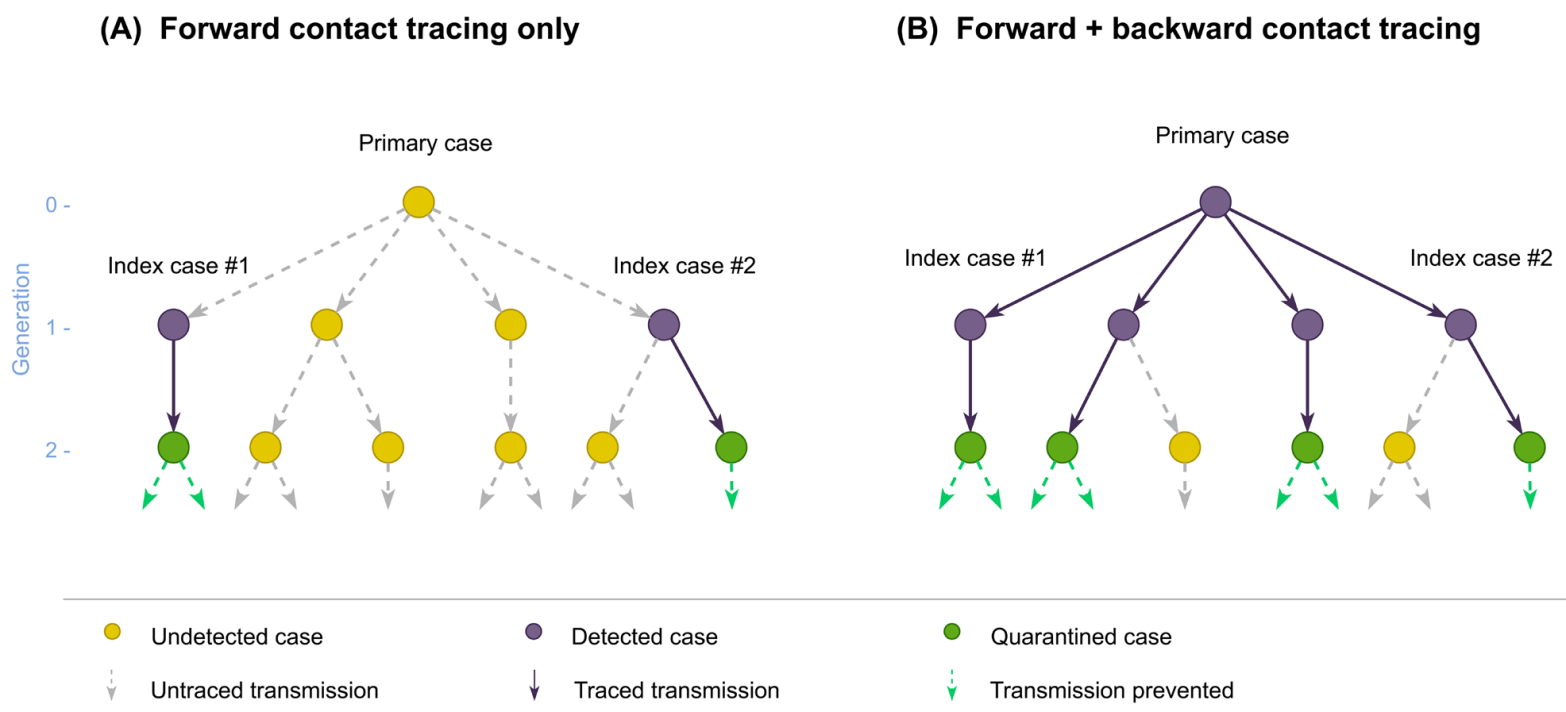
Schematic illustration of forward and backward contact tracing. Two cases (index cases #1 and #2) from a transmission tree originating from an (initially) undetected primary case are assumed to be detected by surveillance. Possible results of contact tracing are shown where ( **A**) only forward tracing is performed or ( **B**) both forward and backward tracing are performed. Some cases may remain undetected because contact tracing can miss cases.

We used a branching process model to compare the performance of forward and backward contact tracing triggered by an index case found by symptom-based surveillance ( Figure 1). We enumerate generations of transmission chains linked to the index case so that the index case belongs to generation-1 (G1). Backward tracing first identifies the primary case (G0) that infected the index case and then applies forward tracing to those infected by the primary case (G1). We represent the transmission chains of COVID-19 by a branching process where *p*( *x*) denotes the offspring distribution, i.e. the probability mass function of the number of secondary transmissions caused by a single case. If an individual is identified as a primary case, they are more likely to have generated more cases than any random case because the probability that a primary case is identified is proportional to the number of cases it generates. Therefore, the number of offspring of the identified primary case follows $p(x|G_{0})=\frac{xp(x)}{E(x)},$ where $E(x)=\sum_{x=0}^{\infty }xp(x).$ The mean number of G1 cases able to be identified by backward tracing (including the index case) is $E(x|G_{0})=\sum_{x=0}^{\infty }x\frac{xp(x)}{E(x)}=\frac{E(x^{2})}{E(x)}=R(1+v^{2}),$ where ( *x*) = *R* is the reproduction number and *v* is the coefficient of variation (the standard deviation of *x* divided by its mean). With a high overdispersion (large *v*), backward tracing of the index case can substantially increase the number of G1 cases to trace. Conversely, the mean number of cases that can be identified by forward tracing is *R* regardless of the degree of overdispersion.

 When we assume *p*( *x*) follows a negative-binomial distribution ^11, 15^ with an overdispersion parameter *k*, backward tracing on average identifies $E(x|{\text{G}}_{0})=R(1+v^{2})=1+R(1+\frac{1}{k})$ G1 cases. Existing studies suggest *k* for SARS-CoV-2 transmission is small and likely to lie within the range of 0.1–0.5 ^11, 16, 17^. A small *k* indicates that the primary case identified through backward tracing typically generates more secondary cases than does a randomly selected case (i.e. ( *x*|G _0_) > *E*( *x*) = *R*).

The higher probability of identifying a large cluster by backward tracing can also be demonstrated by looking at the tail probability of the offspring distribution. Given a negative-binomial offspring distribution $p(x,R,k)=\left(\begin{array}{c} x+k-1\\ x \end{array}\right){\left(\frac{R}{R+k}\right)}^{x}{\left(\frac{k}{R+k}\right)}^{k},$ the probability that a cluster of secondary infections caused by a G0 case identified by backward tracing has a size of *X* or larger is

$$\begin{array}{l} \sum_{x=X}^{\infty }p(x|G_{0})=\frac{1}{R}\sum_{x=X}^{\infty }xp(x;R,k)=\sum_{x=X}^{\infty }\left(\begin{array}{c} x+k-1\\ x-1 \end{array}\right){\left(\frac{R}{R+k}\right)}^{x-1}{\left(\frac{k}{R+k}\right)}^{k+1}\\ \;=\sum_{x=X-1}^{\infty }p(x;\frac{k+1}{k}R,k+1). \end{array}$$

For different combinations of the reproduction number *R* and overdispersion parameter *k*, we estimated the mean size of an identified cluster in backward tracing and the probability of observing a size of at least 5, 10 and 25.

### Simulation of the effectiveness of forward and backward contact tracing

Using our simple branching process model with a negative-binomial offspring distribution, we assessed the potential effectiveness of forward and backward contact tracing. We assumed that contact tracing is triggered by the detection of an index case whose primary case is initially unknown so that our simulation would guide decision making at the operational level (i.e. whether it is worthwhile to implement contact tracing when a case is found). We compared two scenarios: forward tracing only and the combination of forward and backward tracing ( Figure 1). In the forward only scenario, generation-2 (G2) cases resulting from an index case are potentially traced and quarantined; in the combined scenario, more G1 cases can be identified through backward tracing of the primary infection and thus a larger number of G2 cases can be traced and quarantined. As the infectious period of G1 cases is likely to have already passed when they are identified by contact tracing because tracing only starts after the index case is confirmed, we assumed that secondary transmissions caused by G1 cases would not be prevented and that only G2 cases successfully traced could be put in quarantine (which confers a relative reduction *c* in transmission). To account for potential limitations in the effectiveness of contact tracing, we assumed that the primary case is identified with probability *b* and that each offspring of identified cases are traced with probability *q*. G1 cases not traced may be independently found by symptom-based surveillance; we accounted for such independent case finding with a detection probability *d* (although we excluded backward tracing triggered by these cases from analysis), which is expected to be low due to frequent subclinical infections ^18^. All parameters used for simulation are listed in Table 1.

**Table 1.** Parameter notations and values assumed in simulation.

Parameter	Notation	Assumed value in Figure 2 and *Extended data*, Figures S1 and S2 ^19^
Reproduction number	*R*	1.2, 2.5
Overdispersion parameter	*k*	0.2, 0.5
Relative reduction in transmission due to quarantine	*c*	0.2 – 1.0
Probability of identifying the primary (G0) case by backward tracing	*b*	0.5, 0.8
Probability of identifying each offspring of an already identified case	*q*	0.0– 1.0
Probability of a G1 case identified by surveillance independently of contact tracing	*d*	0.1, 0.2, 0.5

We estimated the expected number of generation-3 (G3) cases averted and defined the effectiveness of contact tracing by the relative reduction in the total number of G3 cases. Assuming a negative-binomial branching process with a mean *R* and overdispersion parameter *k*, the mean total number of G3 cases given an index case found by surveillance is

$$C_{3}=\sum_{x_{0},x_{1},x_{2}=0}^{\infty }x_{0}x_{1}x_{2}p(x_{0}|G_{0})p(x_{1})p(x_{2})=E(x|G_{0})E{\left(x\right)}^{2}=R^{2}\left(1+R\left(1+\frac{1}{k}\right)\right).$$

In the forward only scenario, the expected number of G1 cases excluding the initially found index case is $E(x|G_{0})-1=R\left(1+\frac{1}{k}\right),$ of which proportion *d* is independently detected by symptom-based surveillance. Therefore, the total number of G1 cases targeted by forward tracing (including the index case) is $1+Rd\left(1+\frac{1}{k}\right).$ Of the secondary cases generated by these G1 cases ( *R* cases each on average), proportion *q* are successfully traced, i.e. *Rq*(1+ *Rd*(1+1/ *k*)) G2 cases are traced and asked to quarantine on average. The effective reproduction number of quarantined G2 cases is assumed to be *R*(1- *c*); therefore, the estimated number of G3 cases averted is given as

$$\Delta_{\text{F}}=R^{2}qc\left(1+Rd\left(1+\frac{1}{k}\right)\right).$$

In the combined (forward + backward) scenario, G1 cases can also be detected by backward tracing. Of the mean $R\left(1+\frac{1}{k}\right)$ G1 cases potentially under the scope of backward tracing, a proportion (1 – *d*)(1 – *bq*) will remain undetected either by backward tracing or independent detection. As a result, (1-(1- *d*)(1- *bq*)) *R*(1+1/ *k*) G1 cases are identified on average in addition to the index case, leading to tracing of *Rq*(1+(1-(1- *d*)(1- *bq*)) *R*(1+1/ *k*)) G2 cases. By asking these traced G2 cases to quarantine, G3 cases are expected to be averted by

$$\Delta_{\text{F}+\text{B}}=R^{2}qc\left[1+(1-(1-d)(1-bq))R\left(1+\frac{1}{k}\right)\right].$$

The effectiveness of contact tracing in the forward and combined scenarios are obtained as $\frac{\Delta_{\text{F}}}{C_{3}}$ and $\frac{\Delta_{\text{F}+\text{B}}}{C_{3}},$ respectively. The simulation was implemented in R-3.6.1. The replication code and *Extended data* are reposited on GitHub ( https://github.com/akira-endo/COVID19_backwardtracing) and archived with Zenodo ^19^.

An earlier version of this article can be found on medRxiv (DOI: https://doi.org/10.1101/2020.08.01.20166595).

## Results

### Larger clusters are likely to be detected through backward tracing in the presence of overdispersion

The estimated mean and the tail probabilities of the secondary transmissions caused by a primary case identified via backward contact tracing suggests the potential strength of this tracing approach ( Table 2). With a substantial individual-level variation in the number of secondary transmissions per case, characterised by a small overdispersion parameter *k* of a negative-binomial distribution ranging between 0.1–0.5, backward tracing typically leads to a primary case generating 3–10 times more infections than a randomly chosen case (whose mean defines the reproduction number *R*). The tail probabilities, ranging from 25% to 88% for 5 or more offspring ( Table 2), suggest that backward tracing is likely to find a relatively large cluster (≥5) under the plausible parameter settings. These values are striking because the probability of finding such clusters in forward tracing will be much lower. In a case of *R* = 1.2 and *k* = 0.2, only 6% of random cases results in 5 or more secondary infections, as opposed to 53% of primary cases identified by backward tracing.

**Table 2.** Characteristics of transmissions from a primary case identified by backward contact tracing for different combinations of the reproduction number ( *R*) and overdispersion parameter ( *k*).

Reproduction number ( *R*)	Overdispersion parameter ( *k*)	Mean number of transmissions from primary case (( *x* | G _0_))	Probability ( *x* ≥ 5 | G _0_)	Probability ( *x* ≥ 10 | G _0_)	Probability ( *x* ≥ 25 | G _0_)
0.8	0.1	9.8	67%	39%	7%
	0.2	5.8	49%	18%	0.7%
	0.3	4.5	38%	9%	0.1%
	0.4	3.8	30%	5%	0.02%
	0.5	3.4	25%	3%	0.003%
1.2	0.1	14.2	77%	53%	17%
	0.2	8.2	62%	32%	4%
	0.3	6.2	53%	20%	0.9%
	0.4	5.2	45%	13%	0.2%
	0.5	4.6	40%	9%	0.07%
2.5	0.1	28.5	88%	74%	43%
	0.2	16.0	81%	59%	21%
	0.3	11.8	75%	48%	11%
	0.4	9.8	71%	40%	6%
	0.5	8.5	67%	34%	3%

( *x* | G _0_): the mean number of offspring generated by a primary case identified by backward tracing (G0 case). Note that this is larger than the mean number of offspring of a random case. Probability ( *x* ≥ *n* | G _0_): the probability that the number of offspring generated by a G0 case is *n* or greater.

### Backward tracing typically results in multiple-fold increases in the overall effectiveness of contact tracing

**Figure 2.**
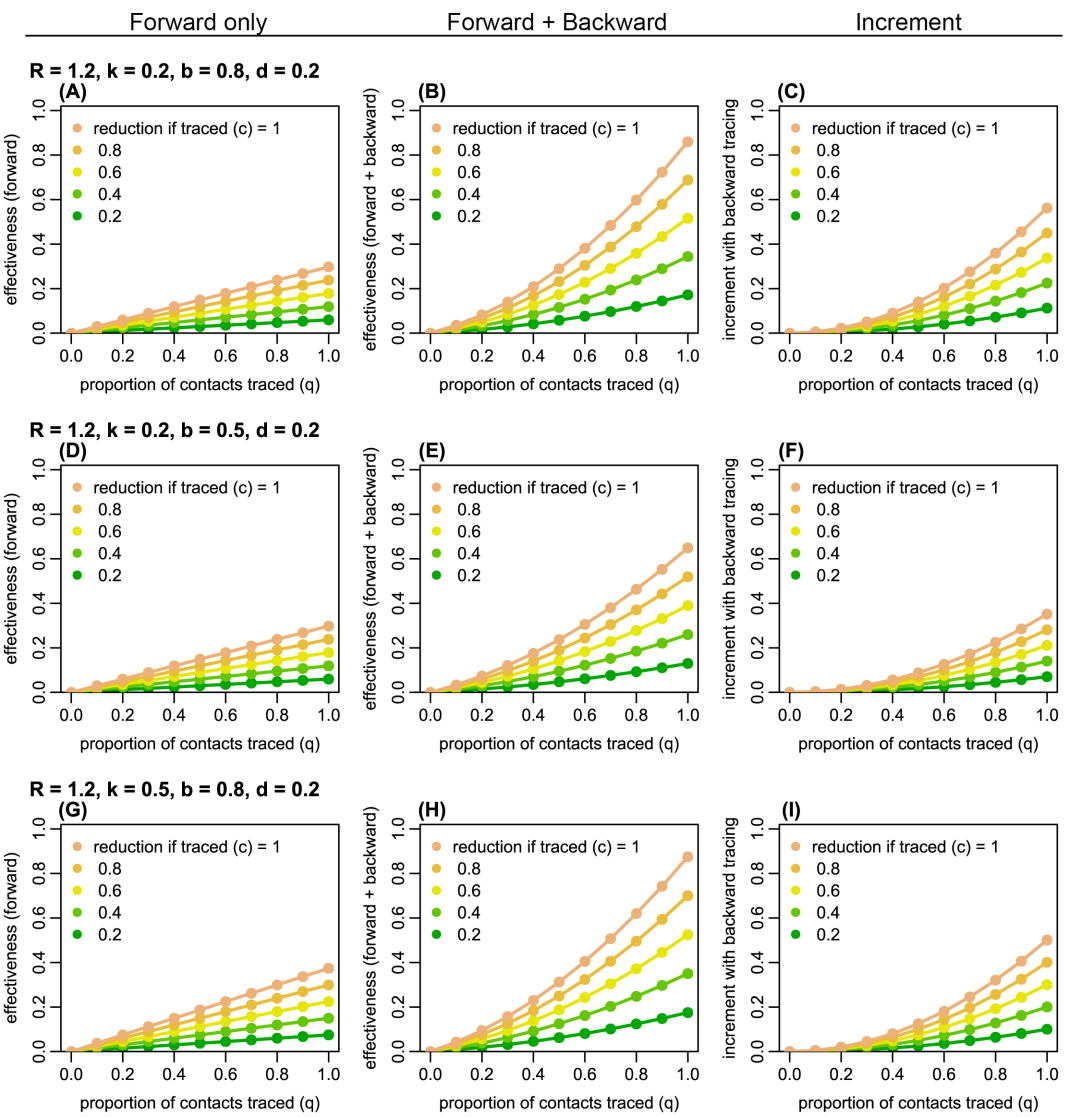
The estimated proportion of generation-3 (G3) cases averted by forward and backward contact tracing for different parameter values. Left panels ( **A**, **D**, **G**): the effectiveness (the proportion of G3 cases averted) of forward tracing alone; middle panels ( **B**, **E**, **H**): the effectiveness of a combination of forward and backward tracing; right panels ( **C**, **F**, **I**): incremental effectiveness by combining backward tracing with forward tracing. Colours represent the relative reduction in transmission from G2 cases if traced and held in quarantine ( *c*). *R*: the reproduction number; *k*: overdispersion parameter; *q*: proportion of secondary infections caused by a detected case successfully traced; *b*: probability of successful identification of the primary case; *d*: probability of detection of generation-1 (G1) cases independent of contact tracing.

**Figure 3.**
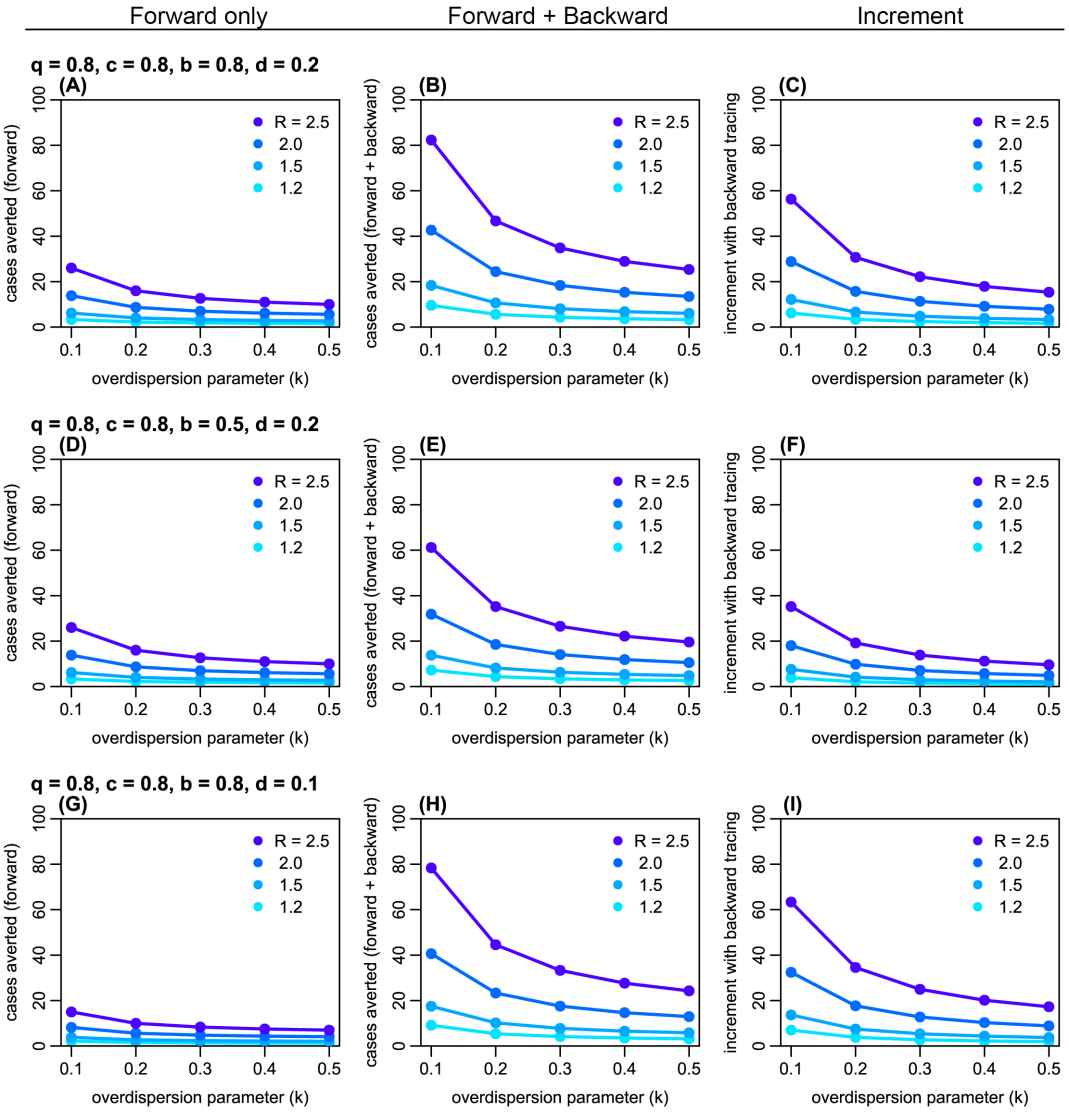
The estimated absolute number of generation-3 (G3) cases averted by forward and backward contact tracing. Left panels ( **A**, **D**, **G**): the number of cases averted by forward tracing alone; middle panels ( **B**, **E**, **H**): the number of cases averted by a combination of forward and backward tracing; right panels ( **C**, **F**, **I**): additional cases averted by combining backward tracing with forward tracing. Colours represent the assumed reproduction number *R*. *k*: overdispersion parameter; *q*: proportion of secondary infections caused by a detected case successful traced; *c*: relative reduction in transmission from quarantined cases; *b*: probability of successful identification of the primary case; *d*: probability of detection of generation-1 (G1) cases independent of contact tracing.

Using a branching process model, we simulated the effectiveness of contact tracing. Across plausible ranges of parameter values, we found that introducing backward tracing in addition to forward tracing increased the effectiveness of contact tracing by a factor of 2–3 ( Figure 2 and *Extended data*, S1 and S2 ^19^). Although the relative improvement in effectiveness by introducing backward tracing is similar between different values of *k* (0.2 and 0.5), the coverage of backward tracing scales up with overdispersion. We found that a higher degree of overdispersion (i.e. small *k*) resulted in a larger absolute number of cases averted by backward tracing ( Figure 3 and *Extended data*, S3). In the presence of substantial overdispersion ( *k* = 0.1), backward tracing is expected to avert 2–3 times more G3 cases than it does in a less-dispersed outbreak ( *k* = 0.5).

## Discussion

Using a simple branching process model, we showed that backward contact tracing has the potential to identify a large proportion of infections because of the observed overdispersion in COVID-19 transmission. For each index cases detected, forward tracing alone can, on average, identify at most the mean number of secondary infections (i.e. *R*). In contrast, backward tracing increases this maximum number of traceable individuals by a factor of 2–3, as index cases are more likely to come from clusters than a case is to generate a cluster. Furthermore, backward tracing contributes to epidemiological understanding of high-risk settings because transmission events with a common source are more likely to be identified. While standard tracing mostly focuses on forward tracing ^3, 4^, there has been increasing interest in a possible combination of forward and backward tracing to control COVID-19 ^14, 20^. Our results provide further evidence for this approach by quantifying the possible benefit of backward tracing, especially when the offspring distribution is highly variable, as is the case with SARS-CoV-2.

There are a number of operational challenges to implementing such contact tracing approaches. Since the number of contacts that lead to transmission is likely to be only a fraction of total contacts experienced by detected cases, expanding the coverage of contact tracing may involve a substantial logistical burden ^21, 22^. Engagement with contact tracing systems and adherence to quarantine may not necessarily reach sufficient levels ^23– 25^. With a longer timeline of contact history to be interviewed, recall bias may affect the success rate of backward tracing. In practice, interviewed cases might be asked not only for specific individuals they know to have contacted but also for a history of locations or events visited, as happens during outbreak investigations so that those who were present can be notified and/or tested. Backward tracing can in effect be viewed as an outbreak investigation process in which new cases and their contacts can be routinely linked via their shared exposure events, supported by cross-referencing over epidemiological, diagnostic and quarantine datasets, with additionally identified infections triggering further tracing. Due to the difficulty in determining the direction of transmission, backward tracing may find a cluster of cases rather than a single primary case. However, our results still apply as long as subsequent forward tracing is conducted for all of the identified cases.

Our model makes some simplifying assumptions. Delays in confirmation and tracing were such that only generation-2 (G2) cases were assumed to be traced and quarantined before becoming infectious. In reality, cases are identified at different points in time and the reduction in infectiousness may be partial if cases are quarantined after becoming infectious (which can be a concern for backward tracing with an additional generation to trace). To allow intuitive comparison, the effectiveness of tracing was measured by the proportion of G3 cases averted given an index case detected by surveillance, and long-term dynamics were not considered. We believe our focus on assessing the effectiveness of a single practice of contact tracing triggered by a detected case is more relevant to operational-level decision making given finite resources. We also did not consider in our model that independently detected multiple index cases may have the same primary case, which can cause duplicated effort of backward tracing. However, such duplication may be minimised if information of each index case is shared among health officials; moreover, overlapping backward tracing still has a benefit because it increases confidence in the identification of primary cases or infection settings.

With these limitations, our results suggest a significant potential benefit to backward tracing, which should be balanced against finite resources. Because backward tracing is operationally a set of forward tracing measures targeting multiple G1 cases in parallel, additional effectiveness requires a proportional amount of effort, in addition to the ‘overhead’ investigation effort to identify other G1 cases. Cost-effectiveness analysis combined with finer-scale dynamic modelling would help further identify the conditions under which backward tracing is most efficient and feasible.

## Data availability

### Underlying data

All data underlying the results are available as part of the article and no additional source data are required.

### Extended data

Zenodo: akira-endo/COVID19_backwardtracing: Implication of backward contact tracing in the presence of overdispersed transmission in COVID-19 outbreaks. https://doi.org/10.5281/zenodo.4062208 ^19^.

Supplementary text ‘COVID19_backwardtracing.html’ contains the following supplementary figures (files stored in subfolder ‘figs’), which are also available on GitHub:

- Figure S1: The estimated effectiveness with R = 2.5.
- Figure S2: The estimated effectiveness with R = 1.2 and d = 0.5.
- Figure S3: The number of generation 3 cases averted with 60% success rate of tracing and 60% relative reduction in transmission during quarantine.

## Software availability

**The reproducible code is available from:** https://github.com/akira-endo/COVID19_backwardtracing.

**Archived repository at time of publication:** https://doi.org/10.5281/zenodo.4062208 ^19^.

**License:** MIT

**Members of the Centre for Mathematical Modelling of Infectious Diseases (CMMID) COVID-19 Working Group (random order)**

Billy J Quilty, Matthew Quaife, Amy Gimma, Charlie Diamond, Rosalind M Eggo, Kiesha Prem, W John Edmunds, Fiona Yueqian Sun, Emily S Nightingale, James W Rudge, Simon R Procter, Rein M G J Houben, Sophie R Meakin, Christopher I Jarvis, James D Munday, Kevin van Zandvoort, Georgia R Gore-Langton, Stéphane Hué, Thibaut Jombart, Damien C Tully, Samuel Clifford, Nicholas G. Davies, Kathleen O'Reilly, Sam Abbott, C Julian Villabona-Arenas, Rachel Lowe, Megan Auzenbergs, David Simons, Nikos I Bosse, Jon C Emery, Yang Liu, Stefan Flasche, Mark Jit, Hamish P Gibbs, Joel Hellewell, Carl A B Pearson, Alicia Rosello, Timothy W Russell, Anna M Foss, Arminder K Deol, Oliver Brady, Petra Klepac

***CMMID COVID-19 Working Group funding statements.*** Billy J Quilty (NIHR: 16/137/109 & 16/136/46), Matthew Quaife (ERC Starting Grant: 757699, B&MGF: INV-001754), Amy Gimma (Global Challenges Research Fund: ES/P010873/1), Charlie Diamond (NIHR: 16/137/109), Rosalind M Eggo (HDR UK: MR/S003975/1, UK MRC: MC_PC 19065), Kiesha Prem (B&MGF: INV-003174, European Commission: 101003688), W John Edmunds (European Commission: 101003688, UK MRC: MC-PC 19065), Fiona Yueqian Sun (NIHR: 16/137/109), Emily S Nightingale (B&MGF: OPP1183986), James W Rudge (DTRA: HDTRA1-18-1-0051), Simon R Procter (B&MGF: OPP1180644), Rein M G J Houben (ERC Starting Grant: #757699), Sophie R Meakin (Wellcome Trust: 210758/Z/18/Z), Christopher I Jarvis (Global Challenges Research Fund: ES/P010873/1), James D Munday (Wellcome Trust: 210758/Z/18/Z), Kevin van Zandvoort (Elrha R2HC/UK DFID/Wellcome Trust/NIHR, DFID/Wellcome Trust: Epidemic Preparedness Coronavirus research programme 221303/Z/20/Z), Georgia R Gore-Langton (UK MRC: LID DTP MR/N013638/1), Thibaut Jombart (Global Challenges Research Fund: ES/P010873/1, UK Public Health Rapid Support Team, NIHR: Health Protection Research Unit for Modelling Methodology HPRU-2012-10096, UK MRC: MC-PC 19065), Samuel Clifford (Wellcome Trust: 208812/Z/17/Z, UK MRC: MC-PC 19065), Nicholas G. Davies (NIHR: Health Protection Research Unit for Immunisation NIHR200929), Kathleen O'Reilly (B&MGF: OPP1191821), Sam Abbott (Wellcome Trust: 210758/Z/18/Z), Rachel Lowe (Royal Society: Dorothy Hodgkin Fellowship), Megan Auzenbergs (B&MGF: OPP1191821), David Simons (BBSRC LIDP: BB/M009513/1), Nikos I Bosse (Wellcome Trust: 210758/Z/18/Z), Jon C Emery (ERC Starting Grant: #757699), Yang Liu (B&MGF: INV-003174, NIHR: 16/137/109, European Commission: 101003688), Stefan Flasche (Wellcome Trust: 208812/Z/17/Z), Mark Jit (B&MGF: INV-003174; NIHR: 16/137/109, NIHR200929; European Commission: 101003688), Hamish P Gibbs (UK DHSC/UK Aid/NIHR: ITCRZ 03010), Joel Hellewell (Wellcome Trust: 210758/Z/18/Z), Carl A B Pearson (B&MGF: NTD Modelling Consortium OPP1184344, DFID/Wellcome Trust: Epidemic Preparedness Coronavirus research programme 221303/Z/20/Z), Alicia Rosello (NIHR: PR-OD-1017-20002), Timothy W Russell (Wellcome Trust: 206250/Z/17/Z), Oliver Brady (Wellcome Trust: 206471/Z/17/Z), Petra Klepac (Royal Society: RP\EA\180004, European Commission: 101003688)
