# Supplementary material for: Implication of backward contact tracing in the presence of overdispersed transmission in COVID-19 outbreaks
Source: Wellcome Open Res. 2021 Mar 31;5:239. Originally published 2020 Oct 13. [Version 3] doi: 10.12688/wellcomeopenres.16344.3 (PMC7610176; doi:10.12688/wellcomeopenres.16344.3)
Supplement: Peer review history

# Peer review: Reviewer response for version 3

**DOI:** 10.21956/wellcomeopenres.18483.r43355

**Published:** 2021-03-31

**Authors:** Edward Hill

**Affiliations:** 1 Zeeman Institute: Systems Biology and Infectious Disease Epidemiology Research (SBIDER), University of Warwick, Coventry, UK; 2 School of Life Sciences, University of Warwick, Coventry, UK; 3 Mathematics Institute, University of Warwick, Coventry, UK

No further comments to make.

Is the work clearly and accurately presented and does it cite the current literature?

Yes

If applicable, is the statistical analysis and its interpretation appropriate?

Yes

Are all the source data underlying the results available to ensure full reproducibility?

Yes

Is the study design appropriate and is the work technically sound?

Yes

Are the conclusions drawn adequately supported by the results?

Yes

Are sufficient details of methods and analysis provided to allow replication by others?

Yes

Reviewer Expertise:

Mathematical epidemiology; infectious disease modelling.

I confirm that I have read this submission and believe that I have an appropriate level of expertise to confirm that it is of an acceptable scientific standard.

# Peer review: Reviewer response for version 2

**DOI:** 10.21956/wellcomeopenres.18216.r42196

**Published:** 2021-01-19

**Authors:** Edward Hill

**Affiliations:** 1 Zeeman Institute: Systems Biology and Infectious Disease Epidemiology Research (SBIDER), University of Warwick, Coventry, UK; 2 School of Life Sciences, University of Warwick, Coventry, UK; 3 Mathematics Institute, University of Warwick, Coventry, UK

The authors have fully addressed the suggestions and I do not have further comments.

Is the work clearly and accurately presented and does it cite the current literature?

Yes

If applicable, is the statistical analysis and its interpretation appropriate?

Yes

Are all the source data underlying the results available to ensure full reproducibility?

Yes

Is the study design appropriate and is the work technically sound?

Yes

Are the conclusions drawn adequately supported by the results?

Yes

Are sufficient details of methods and analysis provided to allow replication by others?

Yes

Reviewer Expertise:

Mathematical epidemiology; infectious disease modelling.

I confirm that I have read this submission and believe that I have an appropriate level of expertise to confirm that it is of an acceptable scientific standard.

# Peer review: Reviewer response for version 1

**DOI:** 10.21956/wellcomeopenres.17971.r40876

**Published:** 2020-11-03

**Authors:** Amy Winter

**Affiliations:** 1 Department of Epidemiology, Bloomberg School of Public Health, Johns Hopkins University, Baltimore, MD, USA

Thank you for the opportunity to review “Implication of backward contact tracing in the presence of overdispersed transmission in COVID-19 outbreaks.” The authors conduct a straightforward branching process simulation to evaluate the additional impact of backwards contact tracing in conjunction with the conventional forward contact tracing. The motivation for the paper builds on our epidemiological understanding of over-dispersion in the transmission of COVID-19, meaning that index cases are disproportionately more likely to result from primary cases that also infected other individuals. As a result, using backwards contact tracing to identify primary cases and their contacts across 2-3 generations expands the potential for contact tracing to identify and isolate more infectious cases beyond forward contract tracing alone. 

 The objectives and motivation are clearly laid out and addressed in the introduction. Figure 1 is a helpful schematic to demonstrate the difference between forward and backwards contact tracing. The methods are straight forward and appropriate to answer the questions at hand. The authors do a nice job evaluating both the proportion and absolute number of generation 3 cases averted across a range of input parameter values. These sensitivity analyses and resulting multi-paneled figures 2, 3, S, S2, & S3 are critical given heterogeneity in populations, their contacts, and the effectiveness of contact tracing and surveillance systems. Lastly, the conclusions are consistent with the results presented. Overall, that paper was well-written and concise. I believe the conclusions add important nuance to the growing scientific literature around contact tracing for COVID-19 and more generally expand the range of interventions available to public health agencies to control the spread of SARS-CoV-2. 

 I do not have any suggested revisions.

Is the work clearly and accurately presented and does it cite the current literature?

Yes

If applicable, is the statistical analysis and its interpretation appropriate?

Yes

Are all the source data underlying the results available to ensure full reproducibility?

Yes

Is the study design appropriate and is the work technically sound?

Yes

Are the conclusions drawn adequately supported by the results?

Yes

Are sufficient details of methods and analysis provided to allow replication by others?

Yes

Reviewer Expertise:

Seroepidemiology, infectious disease modeling, vaccine preventable diseases, demography.

I confirm that I have read this submission and believe that I have an appropriate level of expertise to confirm that it is of an acceptable scientific standard.

# Response

**Published:** 2020-12-18

**Authors:** Akira Endo

**Affiliations:** London School of Hygiene & Tropical Medicine, UK

We thank the reviewer for offering precious time and insightful feedback. We are also grateful for the reviewer's appreciation of the strengths of our paper.

# Peer review: Reviewer response for version 1

**DOI:** 10.21956/wellcomeopenres.17971.r40878

**Published:** 2020-10-22

**Authors:** Edward Hill

**Affiliations:** 1 Zeeman Institute: Systems Biology and Infectious Disease Epidemiology Research (SBIDER), University of Warwick, Coventry, UK; 2 School of Life Sciences, University of Warwick, Coventry, UK; 3 Mathematics Institute, University of Warwick, Coventry, UK

In the study, the authors use a branching process model to explore the implication of combining backward contact tracing with forward contact tracing for control of COVID-19. They use the model to estimate: (i) the mean size of an identified cluster in backward contact tracing; (ii) the probability of identifying a large cluster by looking at the tail probabilities of the offspring distribution; (iii) assess contact tracing effectiveness, measured by generation-3 (G3) cases averted. With the observed overdispersion SARS-CoV-2 transmission, the findings suggest backward contact tracing can be an effective tool for outbreak control.

 I found the manuscript to be very clear and concise. The study premise is well motivated and the methodological approach is appropriate. Sufficient details are provided, via the manuscript and supporting code, to enable others to undertake a similar analysis.

 Please find below additional line-by-line comments: 

**Abstract**

 Results paragraph: It currently reads “a primary case generating 3-10 times more infections than average”. Would it be fair to explicitly say 3-10 times more infections than a randomly chosen case (average case)?

 Final sentence: Suggest amending “of overdispersion as was observed” to “of overdispersion as is observed”.

**Introduction**

 Para 1, L5-8: The sentence beginning “By identifying…” repeats, in my view, much of the information given in the opening sentence and could be removed.

 Para 2, L9: Amend “upper bound of incubation period” to “upper bound of the incubation period”.

 Para 4, L4: Propose repositioning the phrase “using a simple branching process model” to the first line, following “In the present study”.

**Methods**

 Check the first usage of G2 and G3 and that the acronyms are introduced alongside the full phrase (i.e. generation-2 (G2), generation-3 (G3)).

 Simulation subsection, Para 2, L5: Replace “generation-3” by G3.

 Simulation subsection, Para 4, L3: Add “a” ahead of “proportion (1-d)(1-bq)”.

 Simulation subsection, Para 4, Equation for Δ _F+B_: I think there is a typo with regards to the placement of the *R *term. Should the expression in the square brackets read 1 + (1-(1-d)(1-bq))R(1 + 1/k)?

**Results**

 Para 1, L7: In the passage “distribution ranging 0.1-0.5”, to aid flow of text consider replacing with “distribution ranging between 0.1-0.5”.

 Para 1, L10-13: I believe the percentage range specified for the tail probabilities are specific to the number of offspring generated by a primary case being five or higher (rather than 10 or higher, or 25 or higher)? If so, making that criteria explicit within the text would reduce the risk of misinterpretation.

 Para 2, L2-5: In the description of findings presented in Figures 2, S1, S2, there is a lack of comment on the relationship with the variable *q*.

**Discussion**

 Para 2: To support the discussion on the operational challenges of implementing contact tracing approaches, additional references may be added to reflect how some of the stated issues have been encountered as the pandemic continues. For example, user uptake and retention with digital tracing apps (in multiple nations), and the distinction between complex versus non-complex cases in NHS test and trace in contacts of test positive individuals being successfully identified and reached.

**Figures & Tables**

 Table 1: As Figure S2 uses d=0.5, should the assumed values for d listed be expanded to “0.1, 0.2, 0.5”?

 Table 2: I believe there is scope for an accompanying figure to visualise, for a given value of the overdispersion parameter, how the mean number of transmission from a primary case and the tail probabilities vary with the reproduction number.

 Figure 2 caption title: It may be beneficial to add the study definition of tracing effectiveness into the caption title (e.g. “The estimated proportion of generation-3 (G3) cases averted by forward and backward contact tracing”). This would match the style used in the Figure 3 caption title.

 Figure 2 caption, L4: Add the notation *c *in the sentence “Colours represent…” The subsequent description of *c *(L5-6) can then be removed.

 Figure 2 caption, L5: Replace “successful” with “successfully”.

 Figure 3 caption: The sentence “ *R, *the reproduction number” duplicates the preceding sentence and may be removed.

Is the work clearly and accurately presented and does it cite the current literature?

Yes

If applicable, is the statistical analysis and its interpretation appropriate?

Yes

Are all the source data underlying the results available to ensure full reproducibility?

Yes

Is the study design appropriate and is the work technically sound?

Yes

Are the conclusions drawn adequately supported by the results?

Yes

Are sufficient details of methods and analysis provided to allow replication by others?

Yes

Reviewer Expertise:

Mathematical epidemiology; infectious disease modelling.

I confirm that I have read this submission and believe that I have an appropriate level of expertise to confirm that it is of an acceptable scientific standard.

# Response

**Published:** 2020-12-18

**Authors:** Akira Endo

**Affiliations:** London School of Hygiene & Tropical Medicine, UK

We thank the reviewer for helpful insights and suggestions.

 All the points raised by the reviewer were addressed as suggested, except for the following two comments for which we provide justifications as below.

**Results**

 Para 2, L2-5: In the description of findings presented in Figures 2, S1, S2, there is a lack of comment on the relationship with the variable *q*.

*> We agree with the reviewer that q plays an important role as well as other parameters (such as c) in the estimated effectiveness of forward and backward tracing. However, in this result paragraph we aimed to limit our focus on the role of overdispersion k in backward tracing for clarity and conciseness because it is the main scope of this paper. We believe that although we did not specifically mention q here, its role is made clear in Figure 2, where q serves as the horizontal axis.*

**Figures & Tables**

 Table 2: I believe there is scope for an accompanying figure to visualise, for a given value of the overdispersion parameter, how the mean number of transmission from a primary case and the tail probabilities vary with the reproduction number.

*> We believe that a figure as suggested may be of less relevance to the focus of this paper for the following reasons:*

*- The figure for the mean number of transmissions from a primary case E(x|G0)=1+R(1+1/k)  with a constant k would not be very informative as the relationship is linear. We believe that changing k while holding R constant (as we did in Table 2) is preferable to highlight the role of overdispersion.*

*- The mean number of cases averted shown in Figure 3 is closely related to the mean number of transmissions from a primary case and thus an additional figure as suggested could be redundant (and we believe Figure 3 is more public health relevant).*
